# Effects of a quasi-experimental study of using flipped classroom approach to teach evidence-based medicine to medical technology students

**DOI:** 10.1186/s12909-020-1946-7

**Published:** 2020-01-31

**Authors:** Hsien-Li Huang, Chia-Pei Chou, Steve Leu, Huey-Ling You, Mao-Meng Tiao, Chih-Hung Chen

**Affiliations:** 1 Department of Laboratory Medicine, Kaohsiung Chang Gung Memorial Hospital and Chang Gung University College of Medicine, Kaohsiung, Taiwan; 2 Graduate Institute of Adult Education, National Kaohsiung Normal University, Kaohsiung, Taiwan; 3 Department of Family Medicine, Kaohsiung Chang Gung Memorial Hospital, Chang Gung University College of Medicine, Kaohsiung, Taiwan; 4 Institute for Translational Research in Biomedicine, Kaohsiung Chang Gung Memorial Hospital, Kaohsiung, Taiwan; 5 Department of Medical Laboratory Sciences and Biotechnology, Fooyin University, Kaohsiung, Taiwan; 6 Department of Pediatrics, Kaohsiung Chang Gung Memorial Hospital, Chang Gung University College of Medicine, Kaohsiung, Taiwan; 7 Divisions of General Medicine, Department of Internal Medicine, Kaohsiung Chang Gung Memorial Hospital and Chang Gung University College of Medicine, No.123, Dapi Road, Niaosong District, Kaohsiung, 83301 Taiwan

**Keywords:** Quasi-experiment, Evidence-based medicine, eBook, Flipped classroom, Fresno test, Medical technology

## Abstract

**Background:**

Flipped classroom is known to improve learning efficiency and to develop one’s ability to apply high-level knowledge. To investigate the effect of flipped classroom approach on teaching evidence-based medicine to medical technology students, we conducted a tailor-made six flipped classroom based EBM courses for medical technology students.

**Methods:**

This study adopted a qusai-experimental design with 62 medical technology interns as the research object. Students in the experimental group attended the flipped classroom course, while students in the control group attended the traditional course. The learning outcomes were evaluated by Fresno test in both groups. Furthermore, to understand student’s perceptions on the flipped classroom approach, students in the experimental group were required to fill in a satisfaction survey and answer some open-ended questions.

**Results:**

The Fresno test scores of the experimental group were significantly higher than that of the control group. From the results of the satisfaction survey, we know that students were satisfied with this course format. Students claimed that the flipped classroom approach could improve their learning efficiency and the interactions with teacher could help them to think more deeply.

**Conclusions:**

To conclude, most students showed positive attitudes and views on flipped classroom strategy. Moreover, students’ questions were solved more effectively during class resulting in an improvement of effectiveness of evidence-based medicine trainings.

## Background

Evidence-based medicine (EBM) is a combined knowledge of clinical problems, patient’s values, research evidences and clinical experiences [1]. In 2003, the Institute of Medicine (IOM) has published an article, health professions education: A bridge to Quality, in which 5 core competencies in reconstruction of medical professional education are presented. The 5 core competencies include patient-centered care, medical team operation, EBM training, quality promotion and information system application [2]. As EBM supports the acquisition of evidence in an accurate, clear and knowledgeable way, the implementation of EBM in clinical practice will provide a guideline for selection of the most appropriate patient care based on the best available evidence [3, 4]. Solving clinical problems by applying EBM techniques is the main emphasis in Western medical practice, therefore, all medical staffs in a patient-centered cross-disciplinary medical care team should acquire EBM skill [5].

Studies suggested that it is necessary to provide EBM trainings to medical technologists allowing them to develop problem-solving skills and to integrate knowledge for other medical staffs as inspection consultation or interpretative comments [6]. Currently, several hospitals in Taiwan, the Taiwan Evidence-Based Medicine Association, and the Taiwan Society of Laboratory Medicine are offering EBM courses to medical technologists. The main difficulties in teaching EBM to medical technologists are that most of the courses are traditional lecture-style teaching and there is a limited amount of literature focused on laboratory diagnostics in the medical databases. However, a systematic review of EBM teaching suggests that using a single teaching method can strengthen only theoretical knowledge [7]. When EBM is combined with clinical case practicing as a blended-learning model, a considerable improvement in knowledge, skill acquaintance, and learning attitude is observed [8].

With the rapid development of the Internet, the learning style of learners has been changed. Moreover, teaching is no longer limited by time and location. Thus, teachers are able to utilize varies teaching modes and appropriate methods. For a long period of time, teacher is the center of the teaching style of medical education, in which giving a lecture to a group of learner is the main way of teaching [9]. The teaching content mainly consists of knowledge learning and concept understating. Learners have to attend lectures and study in class. And a large number of repeated exercises and tests are required to enhance learners’ competence of understanding [10]. In this teaching model, there is often a lack of interactions between teachers and learners. Learner often does not actively think leading to poor development of his or her cognitive ability [11].

Flipped classroom, an emerging wave in teaching, is considered as a variant of blended-learning model [12]. Instead of providing traditional in-class lectures, the concept of flipped classroom teaching is to teach the basic concept online and to trigger discussion, problem solving and extended thinking during class [13]. As student-centered learning is the core of flipped classroom teaching, the main task of the teacher is to discuss concepts and constructs with students, but not to give lectures. The success of the flipped classroom is mainly due to the increasing convenience of the Internet network that enables motivated students to learn independently through an online teaching platform [14]. Students who learn from the flipped classroom model are more focused during learning. Both their critical thinking skills and their learning attitude are improved [15]. Furthermore, students who are responsible for their own learning activity become active learners [16, 17]. Through peer feedback and learning by doing, knowledge is translated into experience [18].

Although the flipped classroom teaching method has received a positive reaction in most systematic review and aroused a great interest in medical community [15, 19–21], the application of flipped classroom model to EBM teaching or medical technologist training has been limited. The current EBM teaching focuses on solving treatment problems, which does not meet the training requirements of medical technologists. In this study, aiming to emphasize on medical technology educations, we designed a flipped classroom EBM (FC-EBM) course tailor-made for medical technology students. The learning efficiency of the FC-EBM course and the traditional classroom EBM (TC-EBM) course was evaluated with Fresno test. Furthermore, we tried to understand student’s opinions and attitudes towards the FC-EBM course through analyzing the satisfaction survey completed by students who had attended the FC-EBM course.

## Methods

### Design

Quasi-experimental designs [22] were applied to understand whether the implementation of the flipped classroom approaches affects the learning efficiency of EBM. This study was approved by the ethical committee of the Kaohsiung Chang Gung Memorial Hospital, Taiwan (IRB no. 201600978B0 and no.201700615B0).

### Participants

A total of 62 medical technology students from Kaohsiung Chang Gung Memorial Hospital were enrolled for this study. The control group consisted of 24 students who attended the TC-EBM. The experimental group consisted of 38 students who attended the FC-EBM. All participants had no previous experience in EBM learning. The characteristics of each group are shown in Table 1.

**Table 1:** Characteristics of the Control and Experimental Group

	Control group (TC-EBM) *n* = 24	Experimental group (FC-EBM) *n* = 38	*p* value
Age (Mean ± SD)	20.4 ± 1.3	20.3 ± 1.5	0.703
Gender (n)			0.702
Female	20	33	
Male	4	5	
Educational level (n)			0.626
College	16	23	
University	8	15	

### Intervention

**Fig. 1:**
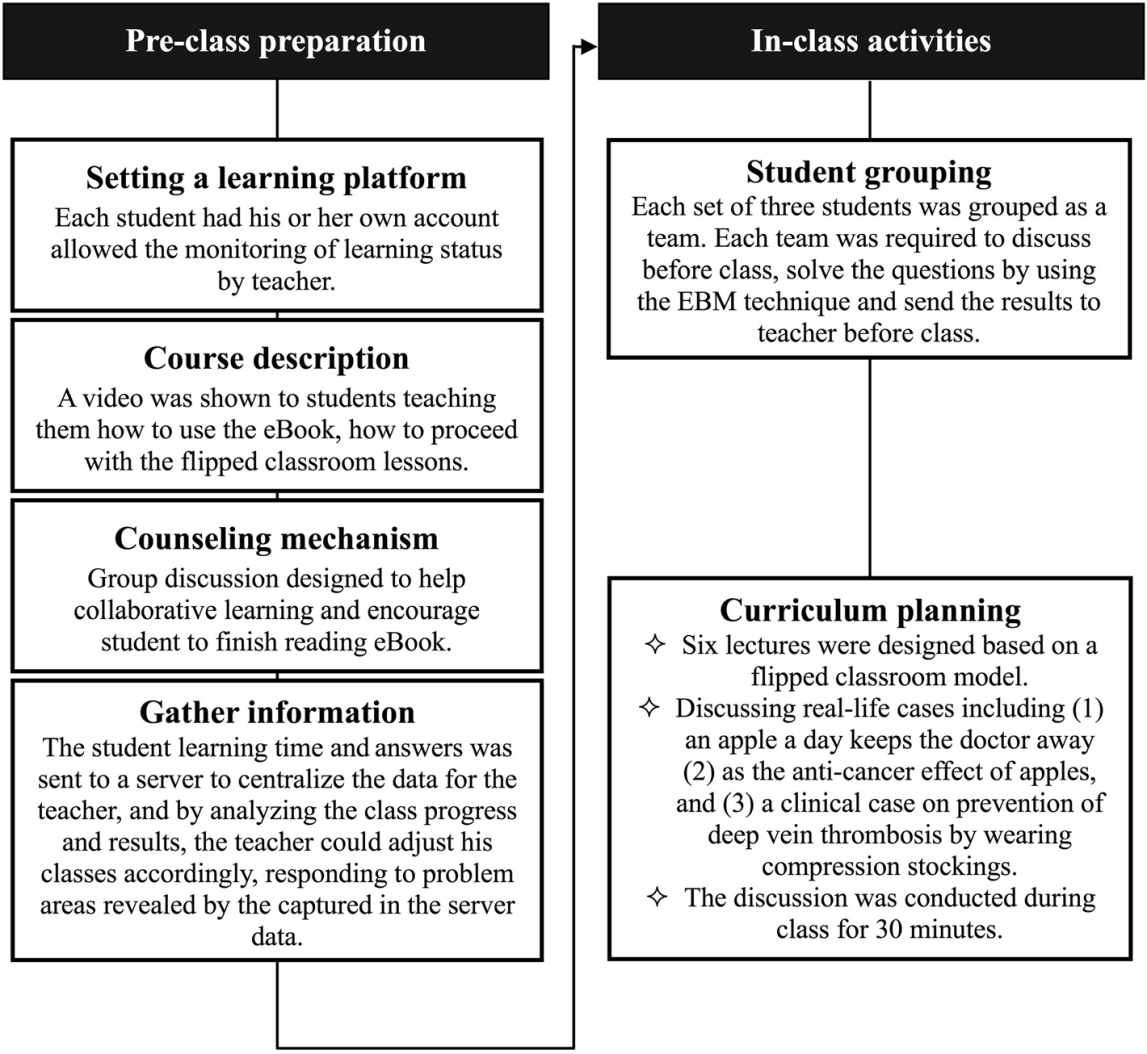
Flowchart of flipped classroom lessons

The class format of the experimental and the control group is illustrated in Table 2. Both groups of students were conducted once a week for 6 weeks and performed the Fresno test 1 week after the end of the course. Since all participants had no experience on attending EBM and flipped classroom course, the design and the procedure of the course were explained before class. Course preparation consisted of the following tasks (Fig. 1): (1) Establishing a learning platform, (2) Course description, (3) Counseling mechanism and (4) Gather information. Activities during class incorporated various components: (1) Student grouping and (2) Curriculum planning.

**Table 2:** Class format for control and experimental group

	Control group (TC-EBM)	Experimental group (FC-EBM)
Prior to class		Read eBook and video material
In class	Standard lectures Q&A session	Problem set Q&A session
After class	Problem set	
Curriculum information		
Teacher	The same	
Course session (total duration)	6 sessions (6 h)	6 sessions (3 h)
No. of student in a group	4~5	2~3
Assessors	Two teachers who did not teach the course (the same teachers in both groups)	
Study cases	4 study cases (1) Does an apple a day really keep the doctor away? (2) Does apple really have an anti-tumor effect? (3) Can wearing compression socks prevent deep vein thrombosis? (4) How to reduce the hemolysis rate?

**Fig. 2:**
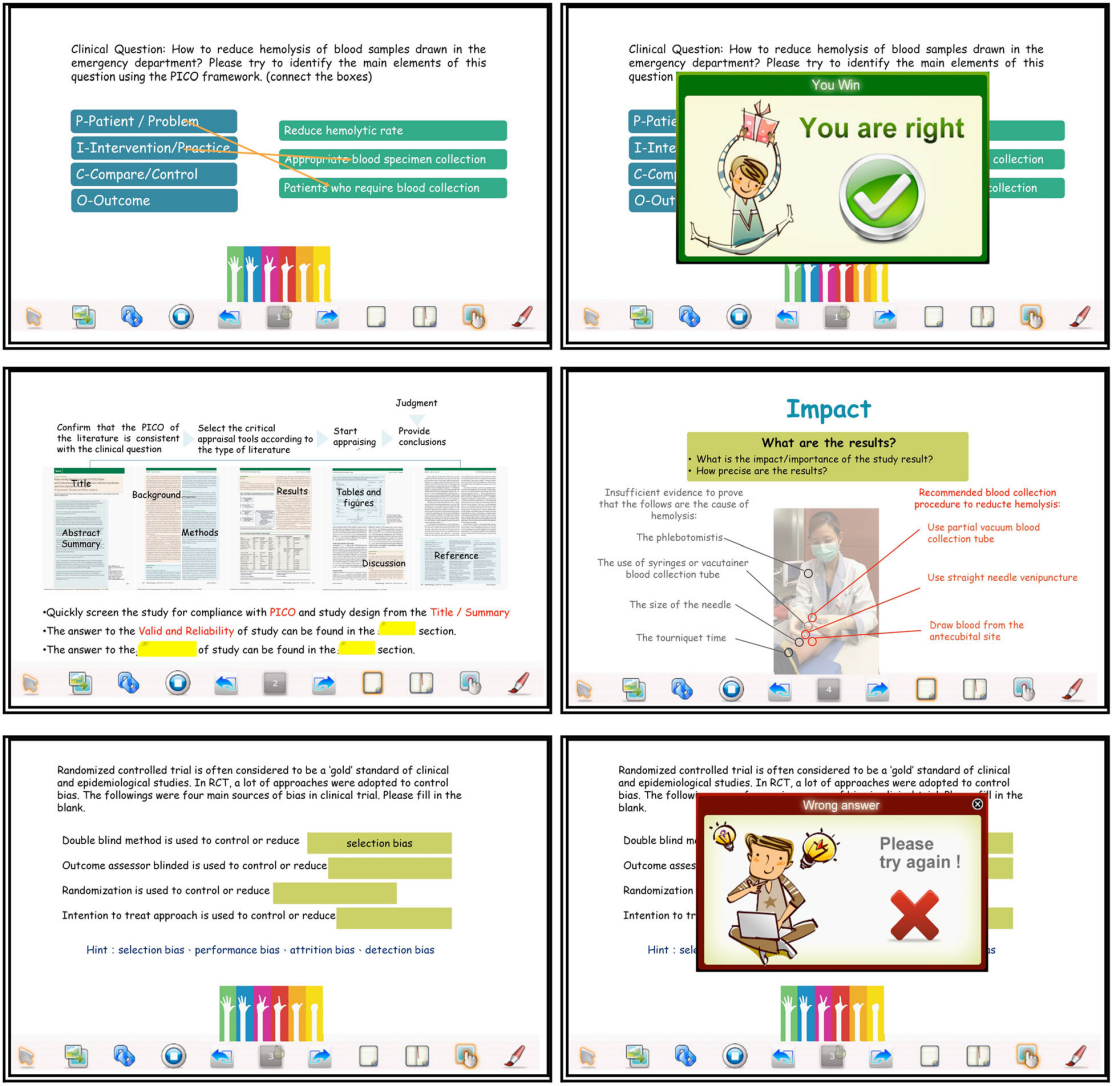
Using SimMAGIC eBook software to create interactive multimedia eBook units of evidence-based medicine

In order to facilitate the reading of students who participated in the flipped classroom course, the contents of the traditional teaching course were streamlined into online self-study eBook (Fig. 2). The case study “reducing the hemolysis rate”, which was discussed in the traditional course, was selected as the main focus of the FC-EBM course [23]. Seven eBook learning units were produced as follows: (1) introduction to EBM, (2) asking questions, (3) finding evidence, (4) type of experiment, (5) reviewing literature, (6) clinical effects and (7) clinical use. The main focus of each eBook unit was summarized and the unit reading time was less than 15 min for student to self-learning at home. Three clinical cases were discussed during class lasted for about 30 min per session.

Each TC-EBM course lasted for at least 1 h per session. The subjects of the traditional teaching courses were (1) basic concepts of EBM, (2) questioning and finding evidence, (3) commonly used research design, (4) reviewing the validity, importance and practice of evaluating literatures and (5) discussing four clinical questions. Problem set was arranged for students who attended the TC-EBM course to practice after class. Student could discuss with teacher in person or through the Line App before class and the teacher would give the answer before next class.

### Instrument

This study adapted Fresno test to evaluate a learner’s knowledge in EBM. The test is a standardized and objective tool to measure one’s competence on the application of EBM [24]. It consists of four domains that include the following: asking clinical question (PICO question), searching strategy, developing critical appraisal skill and applying to clinic. The duration of the test was 30 min. To provide a detail description of the opinions and attitude of implementing flipped classroom to EBM trainings, self-made 14 questions satisfaction survey (Likert 5-point scale) and open-ended questions were used in experimental group.

### Statistical analysis

The demographic data for the control group and the experimental group were examined via chi-square and two-tailed t-tests. Continuous variables were presented as mean ± standard deviation and were analyzed with Mann-Whitney U test. Probability value below 0.05 was considered as statistically significant. All statistical analyses were performed using GraphPad Prism 5.01 (La Jolla, CA).

## Results

### Participant characteristics

The age, gender and education level were similar in both the experimental and control group (Table 1). Most of the participants are college students and most of them are female. Table 2 shows the course timing and curriculum information of the different teaching models. Both groups used the Line app as the information technology. The teaching materials of the flipped classroom course were stored on the digital learning platform, while the teaching materials of the traditional course were supplied in paper format. In terms of course structure and format, the teacher, the assessors, the number of lecture and the study cases were identical in both groups. Only the number of student in each discussion group was different in each group.

### Quantitative findings

**Fig. 3:**
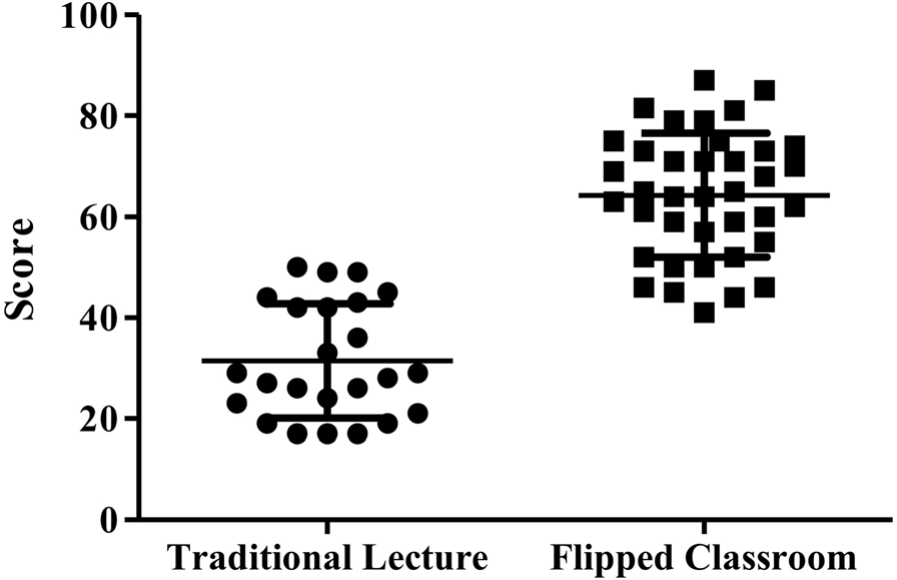
Student’s competence on the application of EBM was scored using Fresno test

Students who attended the flipped classroom-based course (Table 3) had completed all tests and filled in the satisfaction surveys. FC-EBM trained students had a higher Fresno test score than TC-EBM trained students (64.28 ± 12.27 vs 31.46 ± 11.34, *p* <  0.001) (Fig. 3). FC-EBM trained students also had higher average scores on each component of the EBM applications compared to TC-EBM students (Table 4). Of note, the clinical application aspect was again significantly improved (FC-EBM vs TC-EBM: 20.45 ± 6.37 vs 5.54 ± 5.65 vs, p <  0.001).

**Table 3:** Outline of the flipped classroom courses

Lesson 1	Self-study
	Who should we believe? (YouTube video)
	Introduction to evidence based medicine (eBook)
	Asking question (eBook)
	Case discussion
	Does an apple a day really keep the doctor away?
	Does apple really have an anti-tumor effect?
	Can wearing compression socks prevent deep vein thrombosis?
Lesson 2	Self-study
	Acquiring evidence (eBook)
	Embase (Chinese operation booklet)
	Case discussion
	Which PICO is better?
	What are the skills for searching evidence?
	How is the Chinese Electronic Periodical Services important?
	What type of question should be asked?
Lesson 3	Self-study
	Types of experiment (eBook)
	Case discussion
	How to select the right literature?
	Does selected literature provide answers to our question?
	How to match the type of experiment to our question?
Lesson 4	Self-study
	Appraising literature (eBook)
	Case discussion
	Is there any suitable type of research in the literature?
	Is there any missing research in the literature?
	Does literature assess the quality of the research?
Lesson 5	Self-study
	Clinical effectiveness (eBook)
	Case discussion
	Does literature incorporate all suitable research?
	What type of cancer can be prevented by eating apples?
	Do you recommend people to eat apples?
	Does an apple a day really keep the doctor away?
Lesson 6	Self-study
	Clinical application (eBook)
	The development and application of evidence based medicine in the clinic (literature)
	Case sharing
	Pharmacogenomics of adverse drug reactions.
	Rapid bacteria strain identification.
	Drug concentration monitoring.

**Table 4:** Results of Fresno tests

	Control (TC-EBM)	Experimental (FC-EBM)	*p* value
	*n* = 24	*n* = 38	
PICO question (1 question, 10 points)	3.17 ± 3.61	5.68 ± 3.21	0.008
Searching strategy (3 questions, 30 points)	13.75 ± 6.01	19.71 ± 4.77	< 0.001
Critical appraisal (3 questions, 30 points)	9.00 ± 4.12	18.43 ± 5.15	< 0.001
Clinical application (3 questions, 30 points)	5.54 ± 5.65	20.45 ± 6.37	< 0.001

Students who attended the FC-EBM course were satisfied on every aspect listed in the satisfaction survey and scored more than 4 on each item. The satisfaction scores for the interactive eBook, case discussion and flipped classroom-based course were 4.40 ± 0.55, 4.46 ± 0.55 and 4.35 ± 0.62, respectively (Table 5). Among the 14 questions in the satisfaction surveys, the top three most satisfied features were “Teachers provided suitable answers to student’s questions” (4.58 ± 0.50), “Teachers encouraged and provided opportunities for students to ask questions” (4.56 ± 0.53), and “ The content of this course fulfilled the teaching aims” (4.53 ± 0.53).

**Table 5:** Results of the satisfaction survey

Session	Mean ± SD	Questions	Mean ± SD
Interactive eBook (Self-learning at home)	4.40 ± 0.55	Does the content of the eBook include EBM-related information?	4.42 ± 0.53
		Is self-learning from eBook helpful for the EBM course?	4.39 ± 0.58
		Can the content of eBook and video material be applied to discussion in class?	4.39 ± 0.55
Case discussion (During class)	4.46 ± 0.55	Does the case discussion improve the ability of independent thinking?	4.34 ± 0.57
		Does the case discussion result in lively interaction during class?	4.34 ± 0.54
		Did teacher encourage and provide chances for student to ask questions?	4.56 ± 0.53
		Did teacher provide suitable answers to the student’s questions?	4.58 ± 0.50
Flipped classroom (Before and during class)	4.35 ± 0.62	Is the teaching model (eBook and case discussion) properly designed?	4.35 ± 0.58
		Does the teaching model match the teaching goal?	4.53 ± 0.53
		Because of learning from the flipped classroom course, I would tend to solve clinical problems by using EBM.	4.13 ± 0.71
		Is flipped classroom more effective than traditional lecture?	4.34 ± 0.68
		Are you satisfied with this FC-EBM learning model?	4.37 ± 0.55
		Will you expect to continue using the FC-EBM in the future?	4.35 ± 0.60
		Will you recommend other students to attend a FC-EBM course?	4.37 ± 0.63

### Analysis of supplementary open-ended questions

In order to gain a deeper understanding of students’ perceptions and attitudes on flipped classroom, we collected and analyzed the students’ feedback by open-ended questions in the satisfaction survey. Analysis was based on two categories: the effectiveness of such models for learning and the adjustment of classroom environment. Factors that lead to satisfaction include interactive eBooks and in-class case discussions. For example, they mentioned that self-studying the eBook before the start of the course was helpful. The following statements are comments from the students’ feedback (student’s statements are kept unchanged in sentences in italics):

*The contents of the eBook are well organized.*

*Each eBook learning unit is completed in 5-10 minutes. It’s pretty easy for me to study.*

*The eBook included questions related to real life issue which were easy to follow.*

In addition, students mentioned that compared to the monotonous theoretical teaching in traditional teaching, flipped classroom course allowed them to be well prepared before class by self-studying the eBook. In addition, through the active discussion of clinical cases later on class, students had a chance to put the knowledge into practice enhancing their learning effectiveness. In addition, active discussion of clinical cases during class enable students to put their knowledge into practice resulting in improvement of learning effectiveness. Some feedbacks from students are:

*The teacher discussed each question with us. This helped us to clarify some of the content and to comprehend the content better.*

*Through the learning process of the flipped classroom course, questions were solved in a more organized way. Therefore, it was easier for me to solve each question.*

*I gained better understanding of the eBook during class when teacher was discussing with us. When I first read the eBook, I didn’t know how to put into practice. After discussing with the teacher, I was able to relate back to the contents in the eBook.*

Nevertheless, few students found self-study difficult:

*As there were too much attractions at home or in the dormitory, I found it difficult to focus on studying the eBook. The effect of self-study was moderate and I had to read it for more than 2 times to understand the content.*

*EBook do not have to include narrations which may sometimes affect reading. For better reading, only supplementary information could be explained by audio records.*

*Some contents of the eBook are difficult to understand. To have better understanding, I have to search for references.*

## Discussion

In this study, we designed a FC-EBM course focusing on diagnosis problems. Students who participated in the FC-EBM course had significantly higher scores in the Fresno test than the control group. In terms of EBM knowledge, our results show that the teaching strategies and the course design of EBM are in line with the training of medical technologists and are effective for acquisition of knowledge. Flipped classroom strategy is a student-centered active learning technique allowing students to focus on high-level cognitive learning [25]. The implementation of flipped classroom strategy in teaching EBM allows students to develop critical thinking, analyze medical evidences rationally and practically apply their knowledge to clinical care.

These findings were similar to other flipped classroom courses reported by others [26, 27]. Through the theory of Bransford’s science of learning which focuses on the perspective of both teacher and student, we can understand why flipped classroom strategy is effective in learning EBM [28]. This is because flipped classroom strategy engaged students (1) to know new concept and information in advance, (2) to have factual knowledge for concepts understanding, references searching and knowledge application, (3) to apply metacognitive strategies for self-learning through self-monitoring.

Although the classroom time of flipped classroom course is shorter than the traditional teaching, the Fresno test scores of the flipped classroom course are significantly higher than that of the traditional course. The possible reason is that the main focus of flipped classroom strategy is to transmit information, with the shortening of teaching time, the accuracy of learning is increased and the cognitive load level is reduced [29, 30]. In addition, as students know that teacher is monitoring their learning status through the digital platforms, students have the peer pressure that eventually turn to active leaning. This make them more engaged in learning as well. Moreover, flipped classroom is considered as a useful tool for teaching high-risk students as they have longer time to interact with teacher and obtain more helps from powerful peers.

Our quantitative results are further supported by our supplementary open-ended questions feedback that provide insights of student’s perceptions on the flipped classroom strategy. In short, students like the flipped classroom course as both the self-study eBook and the study cases can be explored in depth through frequent teacher-student interactions and peer interactions. In this study, lively cases were discussed allowing students to build knowledge from their past experience and interactions with their group mates. This also helps to develop one’s ability in problem integration, critical thinking and social networking [31].

The results of this study let us believe that medical technology students favored FC-EBM model than TC-EBM model. From the perspective of generations, the possible reason is that all the participants are medical technology interns who belong to the generation Y. In this generation, due to the rapid development of information technology, their learning sources are no longer limited to books. They often conduct learning activities through information and communication technologies such as smart phones or tablets. Thus, our flipped classroom strategy not only changes the teaching and learning activities and the habits of teacher and students, but also meets the learning needs of generation Y.

From the results of the satisfaction survey, it is shows that students are satisfied with acquiring the skill of EBM but they may have low willingness to apply EBM in solving clinical problems. The probable cause is that participants in this study were interns who did not have sufficient clinical experiences. Nieman and colleagues also reported that interns may not fully understand the important of solving clinical problems with EBM methodology [32]. In the future, to strengthen the students’ ability of solving clinical problems, clinical laboratory counseling or questions in patient care from the multidisciplinary medical team should be discussed after the EBM course. These clinical problems are actually the foregrounded questions in EBM learning. Using the skills of EBM, students can be asked to identify relevant literature, discuss issues face-to-face with physicians, and assist physicians and cross-disciplinary teams in solving patient problems.

There were some limitations in this study. First, the experimental setting was not ideal as participants could not be randomly assigned in an educational environment. Second, the number of participants in this quasi-experiment was low. Future use of this teaching model in a larger group is needed to determine whether these results are generally consistent. Third, as teachers and students were participating a research, there might be Pygmalion effect in which student’s performance is affected by teacher’s expectations [33]. Lastly, our course contained only 6 classes in a total of 3 h of training time that was not enough to predict the long-term training performance. Thus, more studies will be needed for long-term tracking of the learning effect and to determine whether the beneficial outcomes of FC-EBM course is sustainable.

In this study, in order to support and strengthen the training of medical technologists, a flipped classroom based EBM course was developed exclusively for medical technology interns. From our analyses, it is known that the use of flipped classroom strategy to train medical technology interns is effective. This not only enhances the learning experience of students, but also introduces many positive effects.

## Conclusion

In this study, we created a EBM courses with 6 sessions implements with a flipped classroom model. According to the analytic results, flipped classroom model can improve the effectiveness of EBM learning of medical technology interns. Most students are satisfied with this teaching method, indicating that this teaching method has been well accepted by students. Yet the duration of the course was short and the number of participant was low. Thus, a long-term study with a larger group of student will be needed to verify our current findings.

## Data Availability

Not applicable.

## Abbreviations

EBM: Evidence-based medicine
FC-EBM: The flipped classroom in teaching evidence-based medicine
PICO: Patient, intervention, comparison, outcome
TC-EBM: The traditional classroom in teaching evidence-based medicine

## References

[1] Rosenberg W, Richardson WS, Sackett DL, Strauss SE, Haynes RB (2000). Evidence-based medicine: how to practice and teach EBM: Churchill Livingstone.

[2] Knebel E, Greiner AC (2003). Health professions education: a bridge to quality: National Academies Press.

[3] Djulbegovic B, Guyatt GH (2017). Progress in evidence-based medicine: a quarter century on. Lancet.

[4] McGinn T, Seltz M, Korenstein D (2002). A method for real-time, evidence-based general medical attending rounds. Acad Med.

[5] Fei J, Li Y, Gao W, Li J (2018). Efficacy of evidence-based medicine training for primary healthcare professionals: a non-randomized controlled trial. BMC Med Educ.

[6] Badrick T (2013). Evidence-based laboratory medicine. Clin Biochem Rev.

[7] Coomarasamy A, Khan KS (2004). What is the evidence that postgraduate teaching in evidence based medicine changes anything? A systematic review. BMJ.

[8] te Pas E, Wieringa–de Waard M, de Ruijter W, van Dijk N (2015). Learning results of GP trainers in a blended learning course on EBM: a cohort study. BMC Med Educ.

[9] Mazur E (2009). Farewell, lecture?. Science.

[10] Myers T, Monypenny R, Trevathan J (2012). Overcoming the glassy-eyed nod: an application of process-oriented guided inquiry learning techniques in information technology. J Learn Des.

[11] Bergmann J, Sams A (2012). Flip your classroom: reach every student in every class every day: international society for technology in education.

[12] Park SE, Howell TH (2015). Implementation of a flipped classroom educational model in a predoctoral dental course. J Dent Educ.

[13] Chao CY, Chen YT, Chuang KY (2015). Exploring students’ learning attitude and achievement in flipped learning supported computer aided design curriculum: a study in high school engineering education. Comput Appl Eng Educ.

[14] Sun X-L. An Action Research Study from Implementing Flipped Classroom Model in Professional English Teaching and Learning. In: 3rd Annual International Conference on Social Science and Contemporary Humanity Development (SSCHD 2017). Paris: Atlantis Press; 2017.

[15] Chen F, Lui AM, Martinelli SM (2017). A systematic review of the effectiveness of flipped classrooms in medical education. Med Educ.

[16] Fautch JM (2015). The flipped classroom for teaching organic chemistry in small classes: is it effective?. Chem Educ Res Pract.

[17] Sun R, Meng R, Wen X (2015). The application of flipped classroom model in TCSL. Lang Teach Linguistic Stud.

[18] Wu W-CV, Hsieh JSC, Yang JC (2017). Creating an online learning community in a flipped classroom to enhance EFL learners’ oral proficiency. J Educ Technol Soc.

[19] Liu Y-Q, Li Y-F, Lei M-J, Liu P-X, Theobald J, Meng L-N, Liu T-T, Zhang C-M, Jin C-D (2018). Effectiveness of the flipped classroom on the development of self-directed learning in nursing education: a meta-analysis. Frontiers Nurs.

[20] Tan C, Yue W-G, Fu Y (2017). Effectiveness of flipped classrooms in nursing education: systematic review and meta-analysis. Chin Nurs Res.

[21] van Alten DC, Phielix C, Janssen J, Kester L. Effects of flipping the classroom on learning outcomes and satisfaction: a meta-analysis. Educ Res Rev. 2019;28:1–18.

[22] Harris AD, McGregor JC, Perencevich EN, Furuno JP, Zhu J, Peterson DE, Finkelstein J (2006). The use and interpretation of quasi-experimental studies in medical informatics. J Am Med Inform Assoc.

[23] Hsiao C-C, Tiao M-M, Chen C-C (2016). Using interactive multimedia e-books for learning blood cell morphology in pediatric hematology. BMC Med Educ.

[24] Tsai J-M, Wu Y-H, Yu S, Li J-Y, Buttrey MJ (2014). Validated Chinese translation of the Fresno test for evidence-based health care training. Int J Gerontol.

[25] Gilboy MB, Heinerichs S, Pazzaglia G (2015). Enhancing student engagement using the flipped classroom. J Nutr Educ Behav.

[26] Deslauriers L, Schelew E, Wieman C (2011). Improved learning in a large-enrollment physics class. Science.

[27] Gillispie V (2016). Using the flipped classroom to bridge the gap to generation Y. Ochsner J.

[28] Council NR (2000). How people learn: brain, mind, experience, and school: expanded edition: National Academies Press.

[29] Karaca C, Ocak M (2017). Effect of flipped learning on cognitive load: a higher education research. J Learning Teaching Digital Age.

[30] Mattis KV (2015). Flipped classroom versus traditional textbook instruction: assessing accuracy and mental effort at different levels of mathematical complexity. Technol Knowl Learn.

[31] Duit R. Learning in science: from behaviourism towards social constructivism and beyond. Int Handbook Sci Educ. 1998;2:3–25.

[32] Nieman LZ, Cheng L, Foxhall LE (2009). Teaching first-year medical students to apply evidence-based practices to patient care. Fam Med.

[33] Niari M, Manousou E, Lionarakis A (2016). The pygmalion effect in distance learning: a case study at the Hellenic Open University. Eur J Open Distance E-learning.

